# WASh multicentre randomised controlled trial: water-assisted sigmoidoscopy in English NHS bowel scope screening

**DOI:** 10.1136/gutjnl-2020-321918

**Published:** 2020-09-07

**Authors:** Matthew D Rutter, Rachel Evans, Zoe Hoare, Christian Von Wagner, Jill Deane, Shiran Esmaily, Tony Larkin, Rhiannon Edwards, Seow Tien Yeo, Llinos Haf Spencer, Emily Holmes, Brian P Saunders, Colin J Rees, Zacharias P Tsiamoulos, Iosif Beintaris, WASh trial team, Mike Bramble

**Affiliations:** 1 Gastroenterology, University Hospital of North Tees, Stockton-on-Tees, UK; 2 Population Health Sciences Institute, Newcastle University, Newcastle upon Tyne, UK; 3 North Wales Organisation for Randomised Trials in Health, Bangor University, Bangor, UK; 4 Behavioural Science and Health, University College London, London, UK; 5 Hartlepool, UK; 6 Centre for Health Economics & Medicines Evaluation, Bangor University, Bangor, UK; 7 Wolfson Unit for Endoscopy, St Mark's Hospital, London, UK; 8 Gastroenterology, South Tyneside NHS Foundation Trust, South Shields, UK

**Keywords:** colorectal neoplasia, colorectal cancer screening, gastrointesinal endoscopy, clinical trials

## Abstract

**Objectives:**

The English Bowel Cancer Screening Programme invites 55 year olds for a sigmoidoscopy (Bowel Scope Screening (BSS)), aiming to resect premalignant polyps, thus reducing cancer incidence. A national patient survey indicated higher procedural pain than anticipated, potentially impacting on screening compliance and effectiveness. We aimed to assess whether water-assisted sigmoidoscopy (WAS), as opposed to standard CO_2_ technique, improved procedural pain and detection of adenomatous polyps.

**Design:**

The WASh (Water-Assisted Sigmoidoscopy) trial was a multicentre, single-blind, randomised control trial for people undergoing BSS. Participants were randomised to either receive WAS or CO_2_ from five sites across England. The primary outcome measure was patient-reported moderate/severe pain, as assessed by patients on a standard Likert scale post procedure prior to discharge. The key secondary outcome was adenoma detection rate (ADR). The costs of each technique were also measured.

**Results:**

1123 participants (50% women, mean age 55) were randomised (561 WAS, 562 CO_2_). We found no difference in patient-reported moderate/severe pain between WAS and CO_2_ (14% in WAS, 15% in CO2; p=0.47). ADR was 15% in the CO_2_ arm and 11% in the WAS arm (p=0.03); however, it remained above the minimum national performance standard in both arms. There was no statistical difference in mean number of adenomas nor overall polyp detection rate. There was negligible cost difference between the two techniques.

**Conclusion:**

In the context of enema-prepared unsedated screening sigmoidoscopies performed by screening-accredited endoscopists, no difference in patient-reported pain was seen when using either a CO_2_ or WAS intubation technique.

**Trial registration number:**

ISRCTN81466870.

**Significance of this study**

**What is already known on this subject?**

- Bowel cancer is the second most common cause of cancer death in the UK. Bowel Scope Screening (BSS) comprises a one-off sigmoidoscopy at 55 years. In a BSS survey, 39% of patients reported moderate or severe pain. Studies indicate that water-assisted colonoscopy (using water rather than CO_2_ insufflation) may decrease pain and increase adenoma detection rates (ADRs). However, no randomised controlled trial has assessed water-assisted sigmoidoscopy (WAS).

**What are the new findings?**

- Our trial did not show reduced patient pain using WAS for unsedated, enema-prepared screening sigmoidoscopy. Patients can be reassured that irrespective of insertion technique, pain in our study was much lower than previously reported. WAS cost on average 40 pence per person more than a CO_2_ procedure.

**How might it impact on clinical practice in the foreseeable future?**

- There is no need for screening sigmoidoscopists to switch to a WAS technique, nor should national policy be amended. Caution should be given to monitoring ADR if WAS is used in enema-prepared sigmoidoscopies. Further research is required to explain why no difference was seen in pain, and why ADRs (although not overall polyp detection rates nor overall adenoma numbers) were lower, particularly as most trial endoscopists preferred the water-assisted technique.

## Introduction

Bowel cancer is the second most common cause of cancer death in the UK, and is estimated to cost the English National Health Service (NHS) £1.6 billion a year.^1^ Based on a large, UK randomised controlled trial (RCT),^2^ ‘Bowel Scope Screening’ (BSS), comprising a one-off sigmoidoscopy at 55 years, began roll-out in 2013, aiming to invite over 650 000 people each year.

Sigmoidoscopy can be painful due to bowel distension and endoscope ‘looping’. Data from a BSS survey of 43 378 patients showed that 39% of patients report moderate or severe pain.^3^ Maximising patient comfort is important as screening participation will reduce if public opinion is that it is unpleasant and anticipation of pain represents an important barrier to public participation.^4^ BSS success in reducing cancer incidence and mortality depends on optimising public participation: current uptake runs at only 43.7% (BSS data, March 2016), indicating that, with improved uptake, the impact on cancer reduction and mortality could double.

Water-assisted colonoscopy (WAC) involves water infusion during scope insertion, instead of traditional CO_2_ insufflation. Water infusion requires a lower volume (under 1 L) than CO_2_ (10 to 20 L), hence reducing distension and looping. Two different techniques have been described: water immersion (WI) and water exchange (WE).^5^ During WI, water is infused to inflate the lumen during scope insertion, then aspirated during withdrawal. In contrast, WE involves removal of the infused water and any bowel gas during insertion, to minimise luminal distension.^5^ Studies indicate that WAC decreases procedural pain.^6–10^ Some studies also show increased adenoma detection rates (ADRs).^8 11 12^ However, no RCT has assessed water-assisted sigmoidoscopy (WAS), nor has any water-assisted RCT been performed in the UK practice.

We aimed to investigate the effects of WAS in BSS. Our primary aim was to test the hypothesis that WAS would lead to decreased procedural pain, when compared with standard CO_2_-assisted insertion, resulting in better patient experience during BSS. Our key secondary aim was to study whether WAS affected the ADR. We also aimed to investigate the effects of WAS on ADR, other aspects of patients’ experience and technical aspects of sigmoidoscopy. Further, we aimed to assess the cost-effectiveness of the two techniques and to perform a discrete choice experiment to elicit patient preferences during sigmoidoscopy.

## Methods

### Trial design

The WASh trial was a multicentre, prospective, two-armed, randomised, single-blinded trial designed to evaluate the effectiveness of WAS in patients referred for BSS screening.^13^

Our primary outcome measure was patient-reported moderate/severe pain, using the standard BSS Likert scale recorded after their procedure. Our key secondary outcome measure was ADR.

We developed the following trial success criteria—either:

- primary outcome achieved (WAS comfort score statistically superior) and no indication of ADR being inferior (defined as WAS ADR within 3% of control ADR); or
- primary outcome not achieved (but comfort score not statistically inferior) but key secondary outcome achieved (ADR statistically superior)

### Participants

Patients referred for screening sigmoidoscopy through the BSS programme were invited to take part in the study. Patients who had absolute contraindications to sigmoidoscopy, lacked capacity to give informed consent, had a previous distal colonic/rectal resection or were receiving ongoing antithrombotic treatment (excluding aspirin) were ineligible for recruitment into the study.^13^

### Endoscopist training

All procedures were performed by screening-accredited endoscopists trained in WAS. Training comprised a baseline questionnaire and a slide presentation (including videos). Endoscopists then attended a training day, including live demonstrations. Prior to trial commencement, endoscopists were required to have completed a training log of at least 20 WAS procedures to confirm competence.^13^

### Procedures

Procedures were performed with enema preparation and without sedation, as mandated by BSS programme. The key principle of the WAS technique was to keep the lumen as collapsed as possible, thereby concertinaing the sigmoid colon, resulting in a straighter and shorter passage between the rectum and the descending colon, hence reducing the tendency for looping. The technique is described in detail in our published protocol.^13^ The technique comprised turning off the CO_2_ pump then infusing water as required to achieve adequate luminal views as the scope advanced. Suctioning of water/faecal residue and gas was performed as needed. While the technique was ideally performed without any gas insufflation, in common with other trials one or two short blasts of CO_2_ were permitted at the discretion of the endoscopist, but where possible, that insufflated gas was suctioned as soon as feasible, thus adhering to the principle of keeping the colon as collapsed as possible. CO_2_ insufflation was reinstated for extubation, which was identical in both arms, in accordance with standard BSS practice.^13^

### Randomisation and blinding

Participants were allocated to WAS or CO_2_ on a 1:1 ratio using a dynamic adaptive algorithm created by the North Wales Organisation for Randomised Trials in Health Clinical Trials Unit.^14^ Participants were stratified by screening centre, scope diameter (adult or paediatric) and history of hysterectomy (men, women with a hysterectomy or women without a hysterectomy). Endoscopists performing the procedure were not masked to treatment arm but where possible, patients were blinded to treatment. Primary data analysis was conducted by a blinded statistician.

### Outcomes

The primary outcome was participant rated procedural pain on a 4-point Likert scale (dichotomised into a binary measure of none/mild versus moderate/severe). The key secondary outcome was ADR, which was calculated from the sigmoidoscopy procedure alone. Other secondary outcomes included pain rated on a validated visual analogue scale (VAS) post procedure and prior to discharge, polyp detection, mean adenomas per procedure, overall procedure time, extent of insertion, use of Entonox, use of hand pressure, patient position changes, technique conversion rates, use of second enema, quality of mucosal views, looping and other procedure-related data. A patient experience questionnaire was completed 24 hours after the procedure. Participants remained in the trial for 14 days following their procedure, for adverse events identification purposes. We also aimed to define the sigmoidoscopist learning curve of the WAS technique during the training period. A full list of outcomes can be found in the study protocol paper.^13^

### Statistics

A sample of 1100 patients was calculated to provide 80% power at a 5% significance level to detect a difference of 30% between the groups on the primary outcome of pain (none/mild versus moderate/severe); this included 5% attrition (PASS, V.15). We assumed an uptake rate of 20% and thus anticipated needing to invite 5500 people within 18 months.

The primary analysis was performed on an intention-to-treat basis. All statistical tests performed were two-sided using a 5% significance level and 95% CI level. Results of secondary analysis were presented without adjustment for multiple comparisons. Outcomes with under 5% missing observations were conducted as complete case analysis, and if at least 5%, multiple imputation (MI) methods were adopted. The MI model included all factors that were used in the analysis models including allocation group, scope diameter, hysterectomy, history of IBS, trust and diverticulosis. A fully defined statistical analysis plan was written, with review from the independent data-monitoring and trial steering committees, prior to completion of data collection.

Mixed effects regression models were run on scale outcomes and logistic regression models (binary, ordinal or multinomial) for categorical outcomes. Scope diameter, hysterectomy, history of IBS and diverticulosis were entered as fixed effect factors and screening centre as a random effect. All assumptions of fitted models were checked and evaluated to hold. The distribution of mean number of adenoma data was evaluated to be highly dispersed around zero; therefore, analysis using a zero-inflated negative binomial model was conducted.

### Economic evaluation

We evaluated the cost-effectiveness of WAS versus CO_2_ sigmoidoscopy. We measured costs from an NHS perspective, focussing on the direct medical costs of both procedures. Only if we observed a significant difference in effectiveness in terms of the primary outcome measure (pain) were we to progress to a full cost-effectiveness analysis.^15^

## Results

### Recruitment

**Figure 1:**
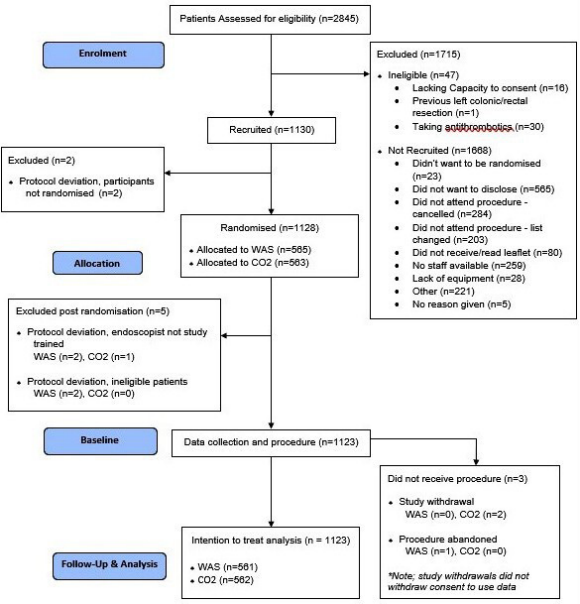
Participant CONSORT flow diagram. CONSORT, Consolidated Standards of Reporting Trials; WAS, water-assisted sigmoidoscopy

Overall, 2845 patients were screened for inclusion in the trial, of whom 1130 were recruited (40%; figure 1) between December 2017 and June 2019. Of the 1130 recruited, two participants were removed from the study before randomisation and following randomisation, a further five participants were removed due to protocol deviations. The final analysis data set therefore comprised 1123 participants (561 WAS; 562 CO_2_). Two patients withdrew from the study following randomisation, however, did not withdraw consent to use their data. No patient was lost to follow-up.

### Missing data

Most of the same-day outcome measures had minimal missing data (below 2%) and complete case analysis was conducted on these. The next-day patient experience questionnaire outcomes had more missing data (24% to 25%); therefore, MI techniques were adopted.

### Demographics

Patient characteristics are detailed in table 1. Participants were 50% men, 43% women without hysterectomy and 7% women with previous hysterectomy. The mean age was 55 years. History of IBS was present in 10% participants. Diverticulosis was not present in 79% of participants, DICA (Diverticular Inflammation and Complication Assessment) grade 1 was present in 17% and DICA grade 2 diverticulosis in 3%.^16^

**Table 1:** Demographics and baseline characteristics

	Overall N (%) (n=1123)	WAS N (%) (n=561)	CO_2_ N (%) (n=562)
Participant age*	55 (0.41)	55 (0.36)	55 (0.45)
Gender			
Men	562 (50)	279 (50)	283 (50)
Women	561 (50)	282 (50)	279 (50)
Hysterectomy			
Women with hysterectomy	81 (7)	43 (8)	38 (7)
Women without hysterectomy	480 (43)	239 (43)	241 (43)
History of IBS			
No	1006 (90)	502 (90)	504 (90)
Yes	117 (10)	59 (10)	58 (10)
Diverticulosis			
No	888 (79)	458 (82)	430 (77)
Grade 1 (<15, no rigidity or no stenosis)	194 (17)	89 (16)	105 (19)
Grade 2 (>15 or rigidity or stenosis)	38 (3)	13 (2)	25 (4)
Missing	3 (<1)	1 (<1)	2 (<1)
Scope diameter			
Adult	986 (88)	492 (88)	494 (88)
Paediatric	137 (12)	69 (12)	68 (12)
Trust			
North Tees and Hartlepool Hospitals NHS Trust	673 (60)	336 (60)	337 (60)
Northumbria Healthcare NHS Foundation Trust	177 (16)	88 (16)	89 (16)
County Durham and Darlington Foundation Trust	132 (12)	68 (12)	64 (11)
London North West Healthcare NHS Trust	80 (7)	39 (7)	41 (7)
South Tyneside Foundation Trust	61 (5)	30 (5)	31 (6)

*Mean (SD) reported.

NHS, National Health Service; WAS, water assisted sigmoidoscopy.

### Procedural pain

When asked immediately following the procedure to reflect on procedural pain, 44% of participants reported no pain, 40% mild, 14% moderate and 2% severe pain. The mean VAS pain score reported immediately after the procedure was 21.1 (SD 22.5; median 10, IQR 3 to 30). Of the 857 (76%) who responded to the next-day survey, 28% recalled no procedural pain, 46% mild pain, 23% moderate and 3% severe pain. Of 854 respondents, 57% felt that the procedure was less painful than expected, 12% felt it was more painful and 31% stated it was as expected. Those who felt the procedure was more painful than expected (12%) were significantly lower than in the national survey (25%) (p<0.01, χ^2^ test).^3^

The results of the logistic (binary) regression analysis (table 2) conducted on the primary outcome of patient-reported moderate or severe pain (judged immediately after the procedure) revealed no statistically significant difference between the WAS and CO_2_ arms (OR=1.13, 95% CI 0.81 to 1.59, p=0.47), with predictive marginal estimates of 14% in WAS and 15% in CO_2_ (table 3). Other patient pain data is included in online supplementary table 1.

10.1136/gutjnl-2020-321918.supp1 Supplementary data

**Table 2:** Results of analysis conducted on study outcomes

Outcome	OR (SE)	95% CI		P value
		Lower	Upper	
Logistic regression				
Pain (none/mild versus moderate/severe) (PO)	1.13 (0.20)	0.81	1.59	0.47
ADR (no, yes) (KSO)	1.45 (0.25)	1.03	2.04	0.03
Pain (none/mild/moderate versus severe)	1.77 (0.87)	0.68	4.62	0.24
Maximum extent of insertion (descending or greater, no further than sigmoid)	1.12 (0.15)	0.87	1.45	0.39
Entonox use (no or yes)	1.32 (0.28)	0.87	2.00	0.19
Need for re-enema (no or yes)	1.32 (0.34)	0.79	2.20	0.28
Need for patient position changes (no or yes)	1.15 (0.18)	0.84	1.55	0.38
Technique conversion (no or yes)	−0.26 (0.09)	0.13	0.52	<0.01
Ordinal logistic regression				
Pain felt 24 hours (none, mild, moderate or severe)	1.21 (0.17)	0.93	1.59	0.15
Pain expected (less painful, as expected or more)	1.23 (0.17)	0.94	1.61	0.13
Embarrassment (none, mild, moderate or severe)	1.02 (0.13)	0.79	1.32	0.86
Satisfaction (very dissatisfied, neither, satisfied or very satisfied	0.87 (0.12)	0.66	1.15	0.33
Repeat procedure (No, not sure or yes)	0.66 (0.26)	0.30	1.44	0.29
Recommend procedure (No, not sure or yes)	1.19 (0.42)	0.59	2.38	0.63
Abdominal pain (none, mild, moderate or severe)	1.33 (0.20)	0.99	1.80	0.06
Nausea (none, mild, moderate or severe)	1.95 (0.78)	0.89	4.27	0.10
Faint feeling (none, mild, moderate or severe)	0.97 (0.28)	0.55	1.71	0.91
Bloating (none, mild, moderate or severe)	1.22 (0.17)	0.92	1.61	0.17
Bottom soreness (none, mild, moderate or severe)	1.07 (0.22)	0.72	1.59	0.75
Soiling (none, mild, moderate or severe)	1.28 (0.36)	0.74	2.23	0.38
Bleeding (none, mild, moderate or severe)	1.50 (0.60)	0.69	3.28	0.31
Sleep disturbance (none, mild, moderate or severe)	0.95 (0.25)	0.57	1.58	0.84
Quality of mucosal views (BBPS) (inadequate, poor, good or excellent)	−0.77 (0.09)	0.61	0.97	0.02
Mixed regression models	**COEF (SE)**			
Patient procedural pain (VAS)	1.59 (1.28)	−0.92	4.11	0.22
Sigmoid insertion time	−0.54 (0.15)	−0.84	−0.24	<0.01
Sigmoid withdrawal time	−0.16 (0.17)	−0.50	0.18	0.35
Overall procedure time	−0.70 (0.24)	−1.17	−0.23	<0.01
Length of scope inserted	−0.04 (0.79)	−1.58	1.50	0.96
Zero-inflated Poisson model				
Mean adenomas per procedure	0.10 (0.18)	−0.26	0.45	0.60

ADR, adenoma detection rate; BBPS, Boston Bowel Preparation Scale; COEF, coefficient; KSO, key secondary outcome; PO, primary outcome; VAS, visual analogue scale; WAS, water-assisted sigmoidoscopy.

**Table 3:** Predictive marginal estimates of PO, KSO and other statistically significant secondary outcomes

Outcome	WAS marginal estimate (SE)	95% CI		CO_2_ marginal estimate (SE)	95% CI	
		Lower	Upper		Lower	Upper
Logistic regression						
PO: pain	0.14 (0.03)	0.08	0.20	0.15 (0.03)	0.09	0.22
KSO: ADR	0.11 (0.02)	0.08	0.15	0.15 (0.02)	0.11	0.20
Ordinal logistic regression						
Quality of mucosal views (BBPS)						
Inadequate	0.02 (0.004)	0.01	0.02	0.02 (0.006)	0.01	0.03
Poor	0.16 (0.03)	0.11	0.21	0.19 (0.03)	0.13	0.25
Good	0.54 (0.02)	0.51	0.58	0.55 (0.02)	0.52	0.59
Excellent	0.28 (0.04)	0.21	0.36	0.24 (0.04)	0.17	0.30
Mixed regression						
Sigmoid insertion time	4.62 (0.47)	3.69	5.55	4.08 (0.47)	3.16	5.01
Overall procedure time	8.83 (1.09)	6.69	10.97	8.12 (1.09)	5.98	10.27

ADR, adenoma detection rate; BBPS, Boston Bowel Preparation Scale; COEF, coefficient; KSO, key secondary outcome; PO, primary outcome; WAS, water-assisted sigmoidoscopy.

### Polyp detection

Of 1123 participants, 290 (26%) had at least one polyp and 113 (10%) had at least one adenoma (online supplementary table 2). Most patients with adenomas (88%) had only one; the most detected being five adenomas in one patient. Logistic regression analysis, detailed in table 2, revealed that the key secondary outcome of ADR was statistically significantly higher in the CO_2_ group (OR=1.45, 95% CI 1.03 to 2.04, p=0.03). The predictive marginal estimates indicate ADR to be 11% in the WAS group, as opposed to 15% in the CO_2_ group (table 3). The mean number of adenomas were 10 per 100 procedures in WAS and 15 per 100 procedures in CO_2_ (NS; not significant). Polyp detection was 143 (25 per 100) in WAS and 147 (26 per 100) in CO_2_ group (NS). Median and IQR for WAS and CO_2_ groups were identical for all polyp sizes (median 3 mm, IQR 2 to 4 mm) and maximal polyp size per patient (median 3 mm, IQR 2 to 5 mm).

### Other procedural outcome measures

Sixty-one per cent of procedures reached at least the descending colon (online supplementary table 2). Retroflexion was performed in 94%. Most procedures did not use Entonox (90%), need a re-enema (94%), external hand pressures (98%) or patient position changes (78%). The most common endoscope model used was the Olympus 260 series (76%). The mean length of scope inserted was 46 cm (SD 14.59).

Eighty per cent of procedures had good or excellent mucosal views on the Boston Bowel Preparation Scale (BBPS).^17^ Mucosal views were statistically inferior in the CO_2_ group (OR=0.77, 95% CI 0.61 to 0.97, p=0.02). Excellent prep was more common in the WAS group, whereas inadequate prep rates were the same in both groups.

The mean overall procedure time was 7.68 (SD 4.30) minutes. After adjustment, those in the WAS group have a longer insertion (4.62 min) and overall procedure time (8.83 min) compared with the CO_2_ group (4.08 min and 8.12 min, respectively; p<0.01 for both).

Overall, 5% of procedures required a technique conversion: significantly more from WAS to CO_2_ (7%) than from CO_2_ to WAS (2%); (OR=0.26, 95% CI 0.13 to 0.52, p<0.01).

All 50 adverse events (in 47 patients) were classed as ‘expected’ and not serious, comprising 21 patients (45%) in the WAS group and 26 patients (55%) in the CO_2_ group (χ^2^ 1 =0.55, p=0.46).

No other outcomes revealed statistically significant differences between WAS and CO_2_ groups. Raw data of pain, procedure-related data and patient experience are presented in the online supplementary tables and analysis results detailed in table 2.

### Other patient questionnaire outcome measures

Of the 856 patients who responded to the question on embarrassment experienced during the procedure, 53% reported none, 39% reported mild, 8% moderate and less than 1% severe embarrassment (online supplementary table 3). Of the 858 patients who rated their experience, 69% were very satisfied, 26% satisfied, 3% neither satisfied or dissatisfied, and 2% dissatisfied. When asked if they would have the same procedure again, 97% said yes, less than 1% said no and just under 3% were unsure. When asked whether they would recommend the procedure to a friend, 97% said yes, under 3% said they were unsure and less than 1% said no (online supplementary table 3).

Of those responding to the questionnaire, 66% reported no abdominal pain or cramps, 28% reported mild, 5% moderate and 1% reported severe abdominal pain or cramps. No bleeding was reported by 97%, mild by 3%, moderate by less than 1% but none reported severe bleeding. No sleep disturbance was reported by 93%, mild by 5%, moderate by 2% and severe by less than 1%. No bloating/wind was reported by 49%, mild by 42%, moderate by 8% and severe by 1%. No bottom soreness was reported by 86%, mild by 13%, moderate by 1% and severe by less than 1%. No soiling was reported by 94%, mild by 5%, moderate by 1% and none reported severe soiling. No nausea/vomiting was reported by 97%, mild by 3%, moderate by less than 1% and severe by less than 1%. No faintness/dizziness was reported by 94%, mild by 6%, moderate by less than 1% and severe by less than 1% (online supplementary table 3).

### Subgroup analyses

Subgroup analyses were conducted on history of IBS, diverticulosis, scope diameter, scope model, extent of procedure, hysterectomy/sex, endoscopist WAS competence prior to study training, study period (first half, second half), BBPS and polyp detection. None aided interpretation of the results with regards to the primary outcome of patient-reported pain. Regarding the key secondary outcome of ADR, the only result of interest was that endoscopists without prior WAS experience had a significantly lower ADR in the WAS arm compared with the CO_2_ arm (no prior experience predictive marginal means ADR 10% in WAS and 15% in CO_2_, p=0.01; prior experience ADR was 21% in WAS and 21% in CO_2_). For prior experience subgroup, the mean number of adenomas were 19 per 100 procedures in both WAS and CO_2_ and for those with no prior experience, the mean adenomas were 8 per 100 procedures in WAS and 14 per 100 procedures in CO_2_. It should be noted that the prevalence of procedures conducted by an experienced endoscopist was low and data for it was not collected a priori for analysis; hence, these results should be interpreted with caution.

### Endoscopist survey

After trial recruitment had finished, the 15 participating endoscopists were asked to complete a questionnaire. Self-reported lifetime experience ranged from 38 to 400 independent water-assisted procedures (median 100), with a success rate ranging from between 60% and 70% to 95% (median 90%). Positive attitude was expressed by 14/15 (93%) towards the water-assisted technique, one was neutral. Ten (67%) preferred the water-assisted technique, one preferred CO_2_ insertion and four were neutral. All stated that they were likely to use the water-assisted technique in their ongoing endoscopy practice.

### Costs of the two procedures

As there was no difference in the primary outcome measure of pain experienced by patients, we assessed the costs of the two procedures rather than performing a full cost-effectivesness analysis. With Reference to NHS costs of £388 for diagnostic flexible sigmoidoscopy with biopsy on an outpatient basis, 19 years and over,^18^ we estimated WAS to be £0.40 more per procedure per patient. The CO_2_ cost £0.03 per litre, whereas water was of slightly higher cost; hence, the difference in price between both procedures.

## Discussion

This is one of the largest RCTs assessing a water-assisted endoscopic technique to be performed to date, and the first in the UK. We believe it is also the first trial to assess the technique in enema-prepared sigmoidoscopy.

### Pain

We found no difference in pain between CO_2_ and WAS—thus, our study proved negative according to its primary outcome measure. This might be because the technique offers no advantage in patient pain, at least in the context of enema-prepared, unsedated sigmoidoscopies performed by screening-accredited endoscopists. Although this is contrary to other trials, our results are plausible as studies to date have all been for colonoscopy rather than sigmoidoscopy. Moreover, the BSS has an endoscopist accreditation process, which may mean the standard technique is higher quality than in most other services. CO_2_ is also mandated in the BSS, whereas in many studies, air was used rather than CO_2_ insufflation: CO_2_ causes less pain than air.^19^ Of note, the overall rates of moderate and severe pain in our trial were significantly lower than in the national survey conducted prior to the trial (p<0.01, χ^2^ test),^3^ perhaps indicating that screening endoscopists have improved their sigmoidoscopy technique since BSS roll-out, or possibly due to the Hawthorne effect (trial sigmoidoscopists’ performance improving due to their knowledge of being observed).

Several other potential explanations were explored. First, it is possible that trial endoscopists were inadequately trained in the water-assisted technique; however, subgroup analysis failed to show any difference in outcome between the first and second half of the study, nor was the outcome different on subgroup analysis of endoscopists with prior experience of the technique. Moreover, after the trial, the majority of endoscopists expressed a preference for water-assisted intubation, implying that they felt they had mastered the technique. While the underlying principle of the water-assisted technique that we taught was to collapse the colon down as much as possible to create a concertinaed sigmoid and straighter path from the rectum to the descending colon, we did not mandate a full water-exchange technique—it is possible that either the full water-exchange technique or a ‘corkscrew hook’ rather than push technique is required to improve comfort.

Second, the technique might have been hampered by inadequate cleansing from enema-preparation; however, no difference in outcome was identified on subgroup analysis limited to cases with good or excellent prep.

Third, most endoscopes did not have a dedicated irrigator channel, potentially hampering the technique; however, subgroup analysis limited to endoscopes with an irrigator channel failed to show a difference.

Fourth, the post-procedure assessment of pain used in our study might have been too insensitive to detect true differences; however, this validated score did reveal a difference between the overall trial pain levels compared with the prior national survey.^3^ Also, no difference was seen using the more sensitive visual analogue score; thus, one can argue that any undetected difference would likely be too subtle to be of clinical significance.

Fifth, unlike in colonoscopy, where the endoscopist must reach a fixed landmark (the caecum), in the BSS, the endoscopist is tasked with intubating as far as is possible within the limitations of the bowel prep and patient comfort—thus, an endoscopist might opt to cease intubation early on if a patient experiences pain, potentially removing a cohort of patients who would have experienced substantial pain with ongoing intubation. We believe this is a likely explanatory factor for the lower levels of pain experienced overall in our trial compared with the earlier BSS survey, where depth of intubation was more than in our trial—it is also possible that there has been a genuine improvement in technique, hence sigmoidoscopy comfort in recent years. Nevertheless, our trial revealed no difference in depth of intubation between the two arms.

Planned subgroup analyses of specific patient cohorts (men/women without hysterectomy/women with hysterectomy, IBS/no IBS, and no diverticulosis/DICA1/DICA2) failed to show any difference in pain between the CO_2_ and water-assisted techniques.

### Polyp detection

In our key secondary outcome measure, ADR was above the minimum BSS performance standard in both arms but was significantly higher in the CO_2_ arm compared with WAS. This could not be explained by any difference in bowel prep quality, nor extubation times. This finding was unexpected, as several prior trials reported higher ADR with a water-exchange intubation technique, although it should be noted that our trial was water-assisted rather than full water-exchange technique. As there was no significant difference between trial arms in the total number of adenomas detected or the polyp detection rate, it is possible that the ADR difference was a chance occurrence. However, it is feasible that the difference was genuine, either due to the technique per se (eg, from reduced detection due to suboptimal views on intubation), a learning curve for the technique (unfamiliarity with the underwater view or distraction by performing the new technique), or the trial protocol (the trial protocol specified that polyps should be removed on extubation where possible). Although one of our subgroup analyses revealed no difference between the first and second half of the trial, another subgroup analysis revealed that the lower ADR was limited to endoscopists without prior experience in the water technique, implying that there may be a longer learning curve for the WAS technique that we had appreciated.

### Cost of WAS and CO_2_ techniques

There was negligible difference in cost between the two procedures.

### Limitations

As with most endoscopy trials, it was not possible to blind the endoscopist to intervention arms, hence there might have been deliberate or inadvertent bias for either CO_2_ or WAS intubation technique. Most patients included in our trial were also recruited at one site (North Tees).

## Conclusion

In conclusion, our trial did not show reduced patient pain using a water-assisted (cf. standard CO_2_-assisted) technique during unsedated, enema-prepared screening sigmoidoscopy performed by screening-accredited endoscopists. Patients can be reassured that irrespective of insertion technique, pain in our study was much lower than previously reported. There is no need for screening sigmoidoscopists to switch to a WAS technique, nor should the BSS programme amend national policy. Caution should be given to monitoring ADR if WAS is used in enema-prepared sigmoidoscopies. Further research is required to explain why no difference was seen in pain, and why ADRs (although not overall polyp detection rates nor overall adenoma numbers) were lower, particularly as most trial endoscopists preferred the water-assisted technique and previous colonoscopy trials have suggested water-assisted techniques were superior.

## Data Availability

Data are available upon reasonable request. All data relevant to the study is included in the article or can be made available upon reasonable request.
