# Supplementary material for: WASh multicentre randomised controlled trial: water-assisted sigmoidoscopy in English NHS bowel scope screening
Source: Gut. 2020 Sep 7;70(5):845–52. doi: 10.1136/gutjnl-2020-321918 (PMC8040154; doi:10.1136/gutjnl-2020-321918)
Supplement: Supplementary data [file gutjnl-2020-321918supp001.pdf]

Supplementary table 1: Patient pain (raw) data results

	Overall N (%) (N = 1123)	WAS N (%) (N =561)	CO2 N (%) (N = 562)
**Patient procedural pain (Likert)**			
	**(N = 1118)**	**(N = 560)**	**(N = 558)**
None	495 (44)	256 (43)	239 (43)
Mild	452 (40)	223 (40)	229 (41)
Moderate	152 (14)	74 (13)	78 (14)
Severe	19 (2)	7 (1)	12 (2)
**How much pain did you feel during the test? (24 hours later)**			
	**(N = 857)**	**(N = 493)**	**(N = 418)**
None	243 (28)	133 (30)	110 (26)
Mild	396 (46)	200 (46)	196 (47)
Moderate	194 (23)	94 (21)	100 (24)
Severe	24 (3)	12 (3)	12 (3)
**Was the test more or less painful than you expected? (24 hours later)**			
	**(N = 854)**	**(N = 437)**	**(N = 417)**
Less painful	486 (57)	257 (59)	229 (55)
As expected	269 (31)	136 (31)	133 (32)
More painful	99 (12)	44 (10)	55 (13)
**Patient procedural pain (VAS)***	**(N = 1118)**	**(N = 560)**	**(N = 558)**
Mean (SD)	21.1 (22.5)	20.3 (22.1)	22.0 (23.0)
Median (interquartile range)	10 (3-30)	10 (2-30)	15 (3-32)

WAS, Water Assisted Sigmoidoscopy; CO2, Carbon dioxide; VAS, Visual Analogue Scale.

Supplementary table 2: Procedural (raw) data

	Overall N (%) (N = 1123)	WAS N (%) (N = 561)	CO2 N (%) (N = 562)
**Did the patient have at least one Adenoma?**			
No	960 (86)	492 (87)	468 (83)
Yes	113 (10)	47 (8)	66 (12)
Missing	50 (4)	22 (4)	28 (5)
**Total number of Adenomas per patient**			
0	960 (86)	492 (87)	468 (83)
1	99 (9)	42 (7)	57 (10)
2	10 (<1)	4 (<1)	6 (<1)
3	2 (<1)	1 (<1)	1 (<1)
4	1 (<1)	0 (0)	1 (<1)
5	1 (<1)	0 (0)	1 (<1)
Missing	50 (4)	22 (4)	28 (5)
**Was a Polyp detected?**			
Yes	290 (26)	143 (26)	147 (26)
No	829 (74)	417 (74)	412 (73)
Missing	4 (<1)	1 (<1)	3 (<1)
**Maximum extent of insertion dichotomised**			
Descending or greater	680 (61)	345 (62)	335 (60)
No further than Sigmoid	440 (40)	215 (38)	225 (40)
Missing	3 (<1)	1 (<1)	2 (<1)
**Entonox use**			
Yes	114 (10)	50 (9)	64 (11)
No	1009 (90)	511 (91)	498 (64)
**Scope Model**			
240	117 (10)	62 (11)	55 (10)
260	852 (76)	428 (76)	424 (75)
290	151 (13)	69 (12)	82 (15)
Missing	3 (<1)	2 (<1)	1 (<1)
**Retroflexion**			
Yes	1055 (94)	532 (95)	523 (93)
No	65 (6)	28 (5)	37 (7)
Missing	3 (<1)	1 (<1)	2 (<1)
**Need for external hand pressures**			
Yes	7 (<1)	2 (<1)	5 (1)
No	1098 (98)	549 (98)	549 (98)
Missing	18 (2)	10 (2)	8 (2)
**Need for patient position changes**			
Yes	231 (21)	107 (19)	124 (22)

Supplementary table 2: Procedural (raw) data

	Overall N (%) (N = 1123)	WAS N (%) (N = 561)	CO2 N (%) (N = 562)
No	872 (78)	442 (79)	430 (77)
Missing	20 (2)	12 (2)	8 (1)
**Need for re-enema**			
Yes	65 (6)	29 (5)	36 (6)
No	1056 (94)	532 (95)	524 (93)
Missing	2 (<1)	0 (0)	2 (<1)
**Quality of mucosal views**			
**Excellent**	276 (25)	148 (26)	128 (23)
**Good**	622 (55)	319 (60)	303 (54)
**Poor**	202 (18)	88 (16)	114 (20)
**Inadequate**	21 (2)	6 (1)	15 (3)
**Missing**	2 (<1)	0 (0)	2 (<1)
**Technique conversion**			
**Yes**	50 (5)	39 (7)	11 (2)
**No**	1070 (95)	521 (93)	549 (98)
**Missing**	3 (<3)	1 (<1)	2 (<1)
**Total Adenomas***	0.12 (0.41)	0.10 (0.34)	0.15 (0.47)
**Length of scope inserted (cm)***	45.6 (14.59)	45.64 (14.51)	45.61 (14.69)
**Overall procedure time (minutes)***	7.68 (4.30)	8.01 (4.35)	7.34 (4.23)

WAS, Water Assisted Sigmoidoscopy; CO2, Carbon dioxide
*Mean (SD) presented

Supplementary table 3: Patient Experience data

	Overall N (%) (N = 1123)	WAS N (%) (N = 561)	CO2 N (%) (N = 562)		Overall N (%) (N = 1123)	WAS N (%) (N = 561)	CO2 N (%) (N = 562)
**How much embarrassment did you feel during the test?**				**Sleep disturbance**			
	**(N = 856)**	**(N=440)**	**(N=416)**		**(N = 857)**	**(N = 440)**	**(N = 417)**
None	456 (53)	237 (54)	219 (53)	None	797 (93)	408 (93)	389 (93)
Mild	333 (39)	167 (38)	166 (40)	Mild	44 (5)	24 (5)	20 (5)
Moderate	65 (8)	36 (8)	29 (7)	Moderate	14 (2)	7 (2)	7 (2)
Severe	2 (<1)	0 (0)	2 (<1)	Severe	2 (<1)	1 (<1)	1 (<1)
**Overall, how satisfied were you with your experience?**				**Bloating/wind**			
	**(N = 858)**	**(N = 440)**	**(N = 418)**		**(N = 856)**	**(N = 440)**	**(N = 416)**
Very dissatisfied	17 (2)	7 (2)	10 (2)	None	420 (49)	222 (50)	198 (48)
Neither satisfied or dissatisfied	27 (3)	12 (3)	15 (4)	Mild	361 (42)	188 (43)	173 (42)
Satisfied	223 (26)	111 (25)	112 (27)	Moderate	68 (8)	29 (7)	39 (9)
Very satisfied	591 (69)	310 (70)	281 (67)	Severe	7 (1)	1 (<1)	6 (1)
**Would you have the same procedure again if needed?**				**Bottom soreness**			
	**(N = 858)**	**(N = 440)**	**(N = 418)**		**(N = 856)**	**(N = 439)**	**(N = 417)**
Yes	830 (97)	423 (96)	407 (97)	None	736 (86)	378 (86)	358 (86)
Not Sure	24 (3)	15 (3)	9 (2)	Mild	112 (13)	59 (13)	53 (13)
No	4 (<1)	2 (<1)	2 (<1)	Moderate	7 (1)	2 (<1)	5 (1)
				Severe	1 (<1)	0 (0)	1 (<1)
**Would you recommend the same procedure to a friend?**				**Soiling**			
	**(N = 857)**	**(N = 440)**	**(N = 417)**		**(N = 856)**	**(N = 440)**	**(N = 416)**
Yes	825 (96)	425 (97)	400 (96)	None	802 (94)	415 (94)	387 (93)
Not Sure	30 (4)	14 (3)	16 (4)	Mild	47 (5)	22 (5)	25 (6)
No	2 (<1)	1 (<1)	1 (<1)	Moderate	7 (1)	3 (1)	4 (1)
				Severe	0 (0)	0 (0)	0 (0)
**Abdominal pain or cramps**				**Nausea/vomiting**			
	**(N = 858)**	**(N = 440)**	**(N = 418)**		**(N = 855)**	**(N = 439)**	**(N = 416)**
None	567 (66)	302 (69)	265 (63)	None	826 (97)	429 (98)	397 (95)
Mild	238 (28)	114 (26)	124 (30)	Mild	23 (3)	7 (2)	16 (4)
Moderate	45 (5)	20 (5)	25 (6)	Moderate	5 (1)	2 (<1)	3 (1)
Severe	8 (1)	4 (1)	4 (1)	Severe	1 (<1)	1 (<1)	0 (0)
**Bleeding**				**Faint feeling/dizziness**			
	**(N = 857)**	**(N = 440)**	**(N = 417)**		**(N = 855)**	**(N = 439)**	**(N = 416)**
None	828 (97)	428 (97)	400 (96)	None	801 (94)	411 (94)	390 (94)
Mild	27 (3)	12 (3)	15 (4)	Mild	48 (6)	25 (6)	23 (6)
Moderate	2 (<1)	0 (0)	2 (<1)	Moderate	4 (<1)	2 (<1)	2 (<1)
Severe	0 (0)	0 (0)	0 (0)	Severe	2 (<1)	1 (<1)	1 (<1)

WAS, Water Assisted Sigmoidoscopy; CO2, Carbon dioxide
